# Epidemiological investigation and genetic evolutionary analysis of PRRSV-1 on a pig farm in China

**DOI:** 10.3389/fmicb.2022.1067173

**Published:** 2022-12-01

**Authors:** Chao Li, Hu Xu, Jing Zhao, Bangjun Gong, Qi Sun, Lirun Xiang, Wansheng Li, Zhenyang Guo, Jinhao Li, Yan-dong Tang, Chaoliang Leng, Jinmei Peng, Qian Wang, Tongqing An, Xuehui Cai, Zhi-Jun Tian, Guohui Zhou, Hongliang Zhang

**Affiliations:** ^1^State Key Laboratory of Veterinary Biotechnology, Harbin Veterinary Research Institute, Chinese Academy of Agricultural Sciences, Harbin, China; ^2^Henan Key Laboratory of Insect Biology in Funiu Mountain, Henan Provincial Engineering Laboratory of Insects Bio-Reactor, China-UK-NYNU-RRes Joint Laboratory of Insect Biology, Nanyang Normal University, Nanyang, China

**Keywords:** PRRSV-1, first detection, main epidemic strain, Chinese pig farm, evolution and genetic diversity

## Abstract

Porcine reproductive and respiratory syndrome virus (PRRSV) has brought serious economic losses to pig industry. PRRSV-1 have existed in China for more than 25 years. The prevalence and features of PRRSV-1 on Chinese farms are unclear. We continuously monitored PRRSV in a pig farm with strict biosafety measures in Henan Province, China, in 2020. The results showed that multiple types of PRRSV coexisted on this single pig farm. PRRSV-1 was one of the main circulating strains on the farm and was responsible for infections throughout nearly the entire epidemic cycle. Phylogenetic analysis showed that PRRSV-1 isolates from this pig farm formed an independent branch, with all isolates belonging to BJEU06-1-like PRRSV. The analysis of selection pressure on ORF5 on this branch identified 5 amino acids as positive selection sites, indicating that PRRSV-1 had undergone adaptive evolution on this farm. According to the analysis of ORF5 of PRRSV-1 on this farm, the evolutionary rate of the BJEU06-1-like branch was estimated to be 1.01 × 10^−2^ substitutions/site/year. To further understand the genome-wide characteristics of PRRSV-1 on this pig farm, two full-length PRRSV-1 genomes representative of pig farms were obtained. The results of amino acid alignment revealed that although one NSP2 deletion was consistent with BJEU06-1, different new features were found in ORF3 and ORF4. According to the above results, PRRSV-1 has undergone considerable evolution in China. This study is the first to report the prevalence and characteristics of PRRSV-1 on a large farm in mainland China, which will provide a reference for the identification and further prevention and control of PRRSV-1.

## Introduction

Porcine reproductive and respiratory syndrome (PRRS) is among the most devastating diseases affecting the pig industry and mainly caused reproductive failure of sows and respiratory symptoms in pigs of all ages (An et al., 2011; Yuzhakov et al., 2017; VanderWaal and Deen, 2018). PRRSV has caused serious economic losses to commercial pig farms in China (Zhang et al., 2022). According to antigenicity, PRRSV isolates can be classified into two separate species, *Betaarterivirus suid 1* (PRRSV-1) and *Betaarterivirus suid 2* (PRRSV-2) which share 60% nucleotide identity at the whole-genome level (Brinton et al., 2021).

Similar to other RNA viruses, PRRSV shows a high evolutionary rate, generating a plethora of variants (Franzo et al., 2022). These mutations accumulate mainly in the NSP2 protein encoded by ORF1a and the GP3, GP4 and GP5 envelope proteins encoded by ORF3-5 (Le Gall et al., 1997; Allende et al., 2000; Chang et al., 2002). The GP5 protein plays an important role in inducing cross protection between virus neutralizing antibody and PRRSV mutation (Kim et al., 2013). Determining the selection pressure for genetic variation of PRRSV by ORF5 is an important part of many molecular evolution studies (Paploski et al., 2021). Moreover, gene recombination events can promote the diversity of PRRSV (Forsberg et al., 2002). Therefore, the long-term investigation of PRRSV infection on a single farm can be helpful for understanding and controlling PRRSV on swine farms (Kim et al., 2011; Li et al., 2021; Xiang et al., 2022).

PRRSV-1 first appeared in Europe and was subsequently reported in Asia, America and other places (Wensvoort et al., 1991; Chen et al., 2011). PRRSV-1 was further classified into four subtypes [subtype 1 (Global), subtype 1 (Russia), and subtypes 2 and 3] based on ORF5 sequences (Stadejek et al., 2006; Shi et al., 2010). Currently, only subtype 1 (Global) has been reported in countries spanning the globe, whereas other subtypes are mainly prevalent in Europe. In Asia, PRRSV-1 has been reported to exist in several countries, including China (Chen et al., 2011). In China, PRRSV-1 was first detected in 1997(Zhang et al., 2020b). Later, PRRSV-1 was sporadically reported around the country, and PRRSV-1 subtype 1 (Global) has been reported in more than 20 provinces in China to date (Zhou et al., 2015; Liu et al., 2017; Chen et al., 2020; Lin et al., 2020; Flay et al., 2022). At present, there are four main clusters of subtype 1 (Global) strains in China: Amervac-like, BJEU06-1-like, HKEU16-like and NMEU09-like (Chen et al., 2017). Recent studies have reported outbreaks of strains of this subtype on Russian pig farms, causing huge economic losses (Havas et al., 2022). Since the emergence of the BJEU06-1-like strain in China in 2006, there have been an increasing number of reports of PRRSV-1 (Chen et al., 2011; Wang et al., 2019; Chen et al., 2020). However, the prevalence and molecular characteristics of PRRSV-1 on Chinese pig farms are unknown. In this study, PRRSV was monitored on a pig-fattening farm, and we studied the epidemic process and molecular characteristics of PRRSV-1 isolates from this pig farm in detail.

## Materials and methods

### Farm information

A pig-finishing farm (500 head) that has been monitored for PRRSV was investigated in the study. The farm is located in Henan Province, China, and there are no neighboring pig farms within 3 km. The 500 piglets (42–49 days old) entered the farm at the same time. Since no cases of PRRSV had been found on this farm, PRRSV-related vaccines had not been administered. A total of 50 serum samples were collected at a ratio of 1:10 from piglets before they were transported to the farm to identify PRRSV antigens by RT-PCR and antibodies by ELISA, and the results were negative for PRRSV antigens, with an 85% antibody-positive rate.

### Sample collection

During the study period, samples were randomly collected in each breeding unit by resident professional veterinarians, and samples (lung, jaw lymph, and blood) were submitted for laboratory testing every 15 days. A total of 132 samples were collected and tested (Supplementary Table S1).

### RNA extraction, PCR screening and sequencing

Tissue sample disposal, RNA extraction, PCR screening and sequencing were conducted as previously described(Xiang et al., 2022). The primers designed to detect PRRSV and to amplify the whole genome are shown in Supplementary Table S2.

### Phylogenetic analysis

Representative PRRSV-1 strains were used as reference strains (Stadejek et al., 2008; Shi et al., 2010; Chen et al., 2017). All data on the reference strains shown in Supplementary Table S3 were downloaded from the NCBI database. All sequences were aligned using MAFFT version 7 in BioAider V1.423 (Katoh and Standley, 2013; Zhou et al., 2020) with the default parameters and were manually adjusted in MEGA6 (Tamura et al., 2013). Phylogenetic trees were constructed as previously described (Jin and Nei, 1990; Ripplinger and Sullivan, 2008; Li et al., 2021).

### Estimation of evolutionary rates

Maximum-likelihood (ML) phylogeny was constructed by IQ-TREE (Nguyen et al., 2015). To ensure sufficient time structures in alignment for reliable rate estimates, we first regressed root-to-tip genetic distances in ML trees using TempEst to determine accurate sampling dates (Rambaut et al., 2016). The collected PRRSV-1 ORF5 sequences were analyzed using the BEAST 1.10.4 package (Kalyaanamoorthy et al., 2017; Zhang et al., 2020a). All analyses were conducted as previously described (Li et al., 2021; Xiang et al., 2022).

### Positive selection pressure analysis

An analysis of the selection pressure acting on the ORF5 codons of PRRSV-1, including 12 new isolates and 129 PRRSV-1 reference strains (Supplementary Table S3), was conducted using the Datamonkey webserver^1^ (Delport et al., 2010).

### Recombinant analysis

The preliminary identification of possible recombination events in our sample sequences was performed using RDP4 (Sharma et al., 2013).

## Results and discussion

### Dynamics of PRRSV on the farm

**Figure 1:**
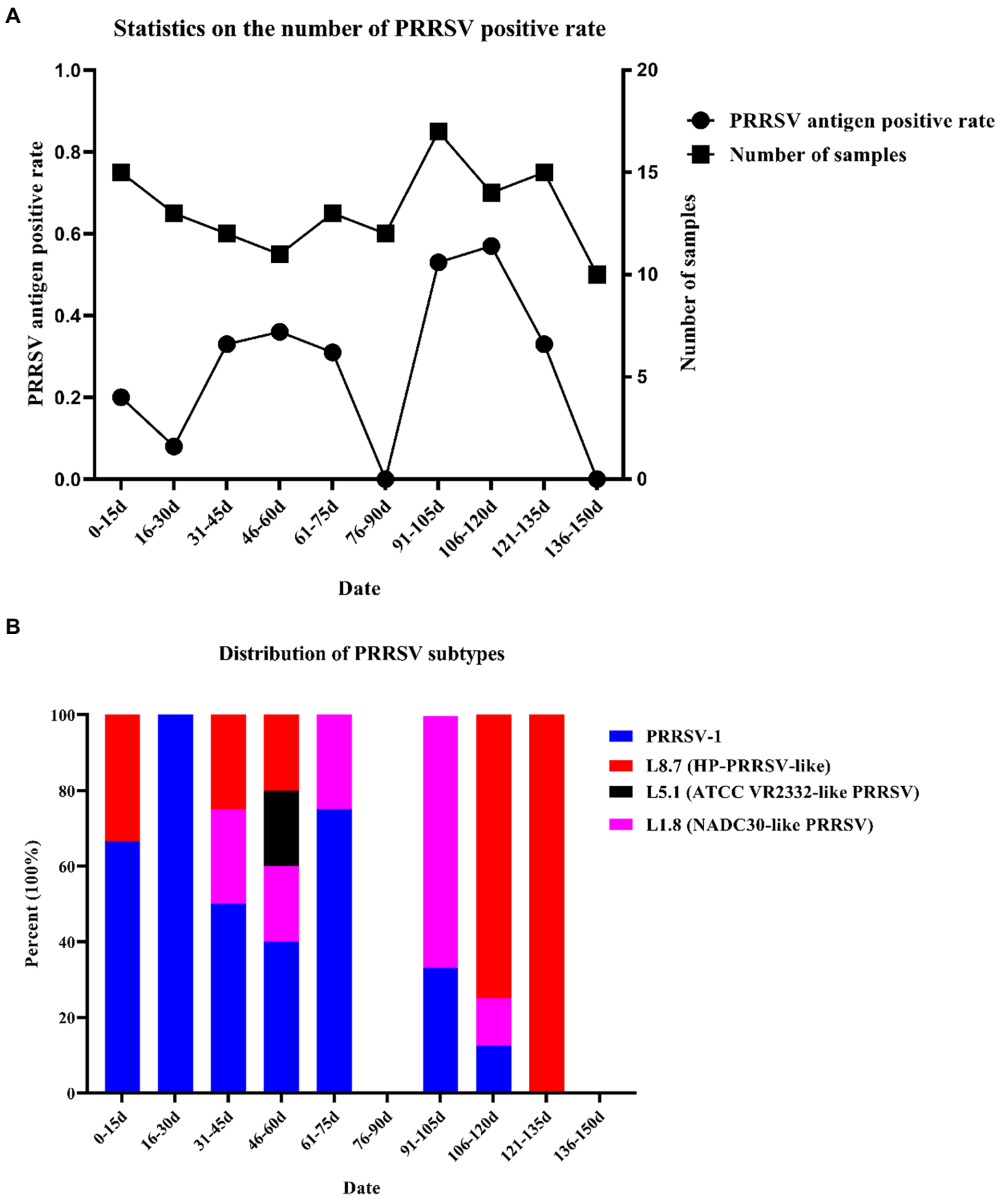
Number of samples collected and statistics of virus subtypes. **(A)** Number of samples tested and number of PRRSV stains detected. **(B)** Distribution of PRRSV subtypes.

A total of 132 samples were collected over 150 days, and samples were sent for testing every 15 days, which was recorded as one time period. Sample details are shown in Supplementary Table S1. After RT–PCR analysis, 38 samples were identified as PRRSV antigen-positive samples, and the positive rate was approximately 28.79%. The presence of PRRSV was detected in eight of the ten time periods, and the highest positivity rate was 57.14% on days 106 to 120 (Figure 1A). To explore the distribution of PRRSV subtypes on this farm, a total of 13 partial NSP2 sequences, 27 ORF5 sequences and 24 partial ORF7 sequences were obtained, and a phylogenetic tree was constructed based on ORF5 and ORF7 (Supplementary Figure S1; Table 1); 14 of these samples were identified as PRRSV-1 (36.94%), 14 as HP-PRRSV-like (36.94%), 10 as NADC30-like PRRSV (26.32%), and 1 as ATCC-VR2332-like PRRSV (2.63%). Therefore, PRRSV-1, HP-PRRSV-like, and NADC30-like PRRSV strains were the main epidemic strains on this farm, which presented multiple types of PRRSV coinfection. The results showed that PRRSV was detected in eight of the ten evaluated time periods, and the presence of PRRSV-1 was detected in seven of these, with the highest percentage of PRRSV-1 positivity consistently found from days 0–75 (Figure 1). The identification of PRRSV-1 as a dominant strain on a Chinese pig-fattening farm is reported for the first time in this study.

**Table 1:** PRRSV identification information.

Number	Sample serial number	PRRSV		
		NSP2	ORF5	ORF7
1	TZJ220	\^a^	\	L8.7 (HP-PRRSV-like)
2	TZJ226	\	PRRSV-1	PRRSV-1
3	TZJ227	\	PRRSV-1	PRRSV-1
4	TZJ234	\	PRRSV-1	PRRSV-1
5	TZJ242	\	L8.7 (HP-PRRSV-like)	\
6	TZJ609	\	PRRSV-1	PRRSV-1
7	TZJ612	\	\	L1.8 (NADC30-like PRRSV)
8	TZJ613	\	PRRSV-1	PRRSV-1
9	TZJ620	L1.8 (NADC30-like PRRSV)	ATCC-VR2332	\
10	TZJ622	\	PRRSV-1	PRRSV-1
11	TZJ623	\	L8.7 (HP-PRRSV-like)	\
12	TZJ624	\	PRRSV-1	PRRSV-1
13	TZJ636	\	L1.8 (NADC30-like PRRSV)	L1.8 (NADC30-like PRRSV)
14	TZJ637	\	PRRSV-1	PRRSV-1
15	TZJ641	\	\	PRRSV-1
16	TZJ642	\	PRRSV-1	PRRSV-1
17	TZJ643			
18	TZJ657	\	PRRSV-1	PRRSV-1
19	TZJ658	\	\	L1.8 (NADC30-like PRRSV)
20	TZJ659	\	PRRSV-1	PRRSV-1
21	TZJ660	\	PRRSV-1	PRRSV-1
22	TZJ661	\	\	L1.8 (NADC30-like PRRSV)
23	TZJ663	\	\	L1.8 (NADC30-like PRRSV)
24	TZJ664	\	\	L1.8 (NADC30-like PRRSV)
25	TZJ669	\	\	L1.8 (NADC30-like PRRSV)
26	TZJ670	\	\	L1.8 (NADC30-like PRRSV)
27	TZJ672	\	\	L1.8 (NADC30-like PRRSV)
28	TZJ675	\	\	PRRSV-1
29	TZJ679	L8.7 (HP-PRRSV-like)	L8.7 (HP-PRRSV-like)	\
30	TZJ680	L8.7 (HP-PRRSV-like)	L8.7 (HP-PRRSV-like)	\
31	TZJ681	L8.7 (HP-PRRSV-like)	L8.7 (HP-PRRSV-like)	\
32	TZJ683	L8.7 (HP-PRRSV-like)	L8.7 (HP-PRRSV-like)	\
33	TZJ684	L8.7 (HP-PRRSV-like)	L8.7 (HP-PRRSV-like)	\
34	TZJ685	L8.7 (HP-PRRSV-like)	L8.7 (HP-PRRSV-like)	\
35	TZJ686	L8.7 (HP-PRRSV-like)	L8.7 (HP-PRRSV-like)	\
36	TZJ687	L8.7 (HP-PRRSV-like)	L8.7 (HP-PRRSV-like)	\
37	TZJ688		L8.7 (HP-PRRSV-like)	\
38	TZJ690	L8.7 (HP-PRRSV-like)	L8.7 (HP-PRRSV-like)	\
39	TZJ692	L8.7 (HP-PRRSV-like)	L8.7 (HP-PRRSV-like)	\

\^a^: Sequence not detected.

Since the first PRRSV strain was reported in China in 1996, PRRSV has been a major threat to Chinese pig farms. Considering the increasing reports of PRRSV-1 across China since 2011 (Chen et al., 2017, 2020), we speculate that coinfection with PRRSV-1 and PRRSV-2 may have profound effects on pig farms. During the course of this study, we discovered an interesting phenomenon: we detected the presence of PRRSV in the first time period (0–15 days) even though the pigs on this farm had never been infected with PRRSV before, and the PRRSV antigen results obtained during the entry of the pigs at this time were negative. Nevertheless, latent PRRSV infection has always been a very troublesome problem, and results similar to those obtained in this study have appeared in previous studies (Kick et al., 2019), in which PRRSV could not be detected in certain time periods. Therefore, we speculate that the piglets were latently infected with PRRSV before they entered the farm.

### Sequence analysis of ORF5 of PRRSV-1

**Figure 2:**
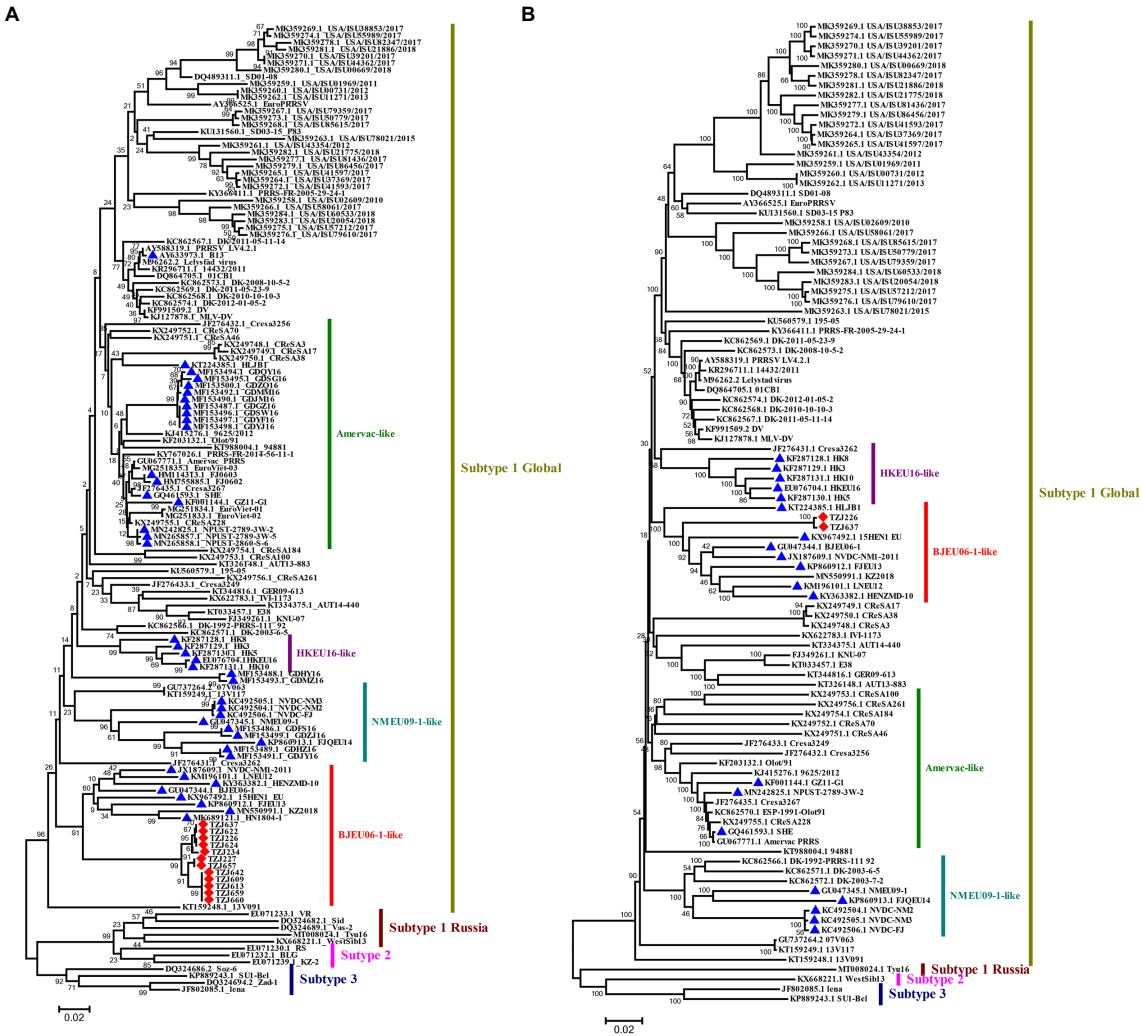
Phylogenetic analysis of PRRSV-1. **(A)** Phylogenetic tree constructed based on the ORF5 gene of PRRSV-1 isolates and reference PRRSV strains from each subtype. **(B)** Phylogenetic tree constructed based on full-length genomes of 2 PRRSV-1 isolates and reference PRRSV strains from each subtype. In the NCBI library, the PRRSV-1 strains from China and the PRRSV-1 strains from the pig farm are marked with ▲ and ◆, respectively. Chinese PRRSV 1 isolates all belong to subtype 1 and can be divided into four subgroups (Amervac-like, BJEU06-1-like, HKEU16-like, and NMEU09-1-like isolates). The scale bars indicate the number of nucleotide substitutions per site.

A total of 12 complete ORF5 sequences of PRRSV-1 were identified. The ORF5 nucleotide homology of the 12 strains was between 97.9–100%. Previous studies have reported that PRRSV-1 can be divided into 4 subtypes, among which only subtype 1 has been found in China, and four groups have ultimately been formed: Amervac-like, BJEU06-1-like, HKEU16-like and NMEU09-like (Chen et al., 2017). To understand the origin and evolution of PRRSV-1 on this farm, the available data on all PRRSV-1 from China and some other representative strains of PRRSV-1 were downloaded from NCBI and compared (Supplementary Table S3). A phylogenetic tree of the PRRSV-1 isolates analyzed in this study was constructed according to a previously reported typing method (Chen et al., 2017), and the results showed that the PRRSV-1 strains on this pig-finishing farm were BJEU06-1-like strains (Figure 2A). BJEU06-1-like strains first appeared in mainland China in 2006, and the spread of these strains in China has been reported several times since then (Chen et al., 2011; Liu et al., 2017; Zhang et al., 2020b). By comparing the homology of ORF5 nucleotide sequences from this branch, it was found that the identity of PRRSV-1 strains from this pig farm with other strains of this branch was between 85.0–88.8% (Supplementary Table S4), representing a reduction in homology compared to the previous results for this branch (87.5–94.2%, Supplementary Table S5). Therefore, the BJEU06-1-like group has been circulating since its emergence in China and has continued to evolve. While early studies suggested that a lower degree of variability might exist in PRRSV-1 (Suarez et al., 1996; Drew et al., 1997), later studies in some countries, such as Denmark and Italy, reported high divergence of PRRSV-1 isolates (Oleksiewicz et al., 2000; Forsberg et al., 2001, 2002). To understand the genetic evolution of PRRSV-1 isolated from this pig farm in China, the ORF5 sequences of PRRSV-1 isolated on this farm were compared with those of PRRSV-1 isolates previously reported in China, and the results showed that the consistency was between 79.9 and 88.8% (Supplementary Table S5). Additionally, the nucleotide sequence identity of BJEU06-1 strains on this pig farm was as high as 97.9–100%, so we speculate that the PRRSV-1 isolates identified on this farm evolved from a single strain.

### Evolution of ORF5 of PRRSV-1 on the farm during the study

The evolution of a single strain on the farm created favorable conditions for estimating the evolutionary time of this type of strain (Li et al., 2021). The PRRSV-1 strains from this farm showed a strong time signal [the correlation (*r*^2^) between the genetic difference and sampling time was 0.28] and were thus suitable for molecular clock-based phylogenetic analysis. The PRRSV-1 isolates showed a lower mutation rate [1.01 × 10^−2^ substitutions/site/year, 95% highest posterior density (3.18 × 10^−3^/ ~ 1.66 × 10^−2^)] than PRRSV-1 isolates from other countries (1.47 × 10^−2^ substitutions/site/year) but a higher mutation rate than PRRSV-2 (9.6 × 10^−3^ substitutions/site/year; Kim et al., 2011; Li et al., 2021; Xiang et al., 2022). The higher evolutionary rate provides a theoretical basis for the differentiation of the BJEU06-1-like branch, and this result suggests that this branch may accumulate many mutations in the next short time period. During the rapid evolution of the virus, natural selection is generally imposed episodically. A growing number of studies have reported the emergence of virulent strains during PRRSV evolution (An et al., 2010; Havas et al., 2022; Yuan et al., 2022), associated with significant economic losses. Therefore, for PRRSV-1 monitoring, more data should be obtained to prevent the occurrence of tragic outcomes.

### Positive selection pressure analysis of ORF5 of PRRSV-1 on this pig farm

The GP5 protein of PRRSV is one of the most important structural proteins exposed on the surface of the virion and contains epitopes involved in virus neutralization and protection (Music and Gagnon, 2010). The identification of point mutations under positive selection pressure is often interpreted as evidence of increased evolutionary fitness (Kryazhimskiy and Plotkin, 2008). Selection pressure analysis of the ORF5 gene revealed four positively selected sites (amino acids 2, 8, 10 and 106) based on at least three methods (Table 2). The identified positively selected sites were diverse, and most of the sites were hydrophilic (Table 3). There were no regular changes in the polarity of the positively selected amino acids, but a change from a nonpolar amino acid (F) at position 10 to a polar amino acid (S) was observed (Table 3). Selection pressure analysis revealed that the ORF5 gene had experienced positive selection, and several positively selected sites were identified, which could help to identify the molecular determinants of virulence or pathogenesis and to clarify the driving force of PRRSV-1 evolution in China.

**Table 2:** Selection pressure analysis of the GP5 protein of PRRSV.

Protein	Codon	SLAC		FEL		MEME		FUBAR	
		dN-dS	*p*	alpha = beta	*p*	*β*+	*p*	*β*-α	Post.pr.
GP5	2	1.07	0.0362	0.111	0.005	0.45	1	0.656	0.973
	8	1.91	0.00182	\	0.7368	1.03	1	1.61	0.995
	10	0.895	0.102	10,268	0.0469	4.45	0.17	0.557	0.938
	106	1.87	0.0718	1.731	0.0301	4.74	0.51	3.853	0.881

The sites found to be under positive selection (significance value) by at least three methods are shown. Codons with *p* < 0.1 by the SLAC method, *p* < 0.1 by the FEL method, *p* < 0.1 by the MEME method, or Post.pr. A value ≥0.9 by the FUBAR method was considered to be under positive selection.

**Table 3:** Positions and polarities of the positively selected amino acids.

Sites	2		8			10	106		
Majority	T^0^		E^−^			S^0^	N^0^		
Substitution	R^+^	K^+^	G^0^	V^×^	A^×^	F^×^	G^0^	R^+^	K^+^

The polarities of positively selected aa are indicated at the top-right corner of each amino acids. ×: Nonpolar amino acids (hydrophobic). +: Positively charged amino acids (hydrophilic). −: Negatively charged amino acids (hydrophilic). 0: Uncharged amino acids (hydrophilic).

### Whole-genome analysis of TZJ226 and TZJ637

To explore the genomic characteristics of PRRSV-1 on this pig farm, the full-length genomes of TZJ226 (the first PRRSV-1 isolate from this farm) and TZJ637 (PRRSV-1 from the fifth of the ten time periods, to analyze whether the full-length PRRSV-1 genome showed variation over a shorter time period) were evaluated in this study. The complete genomes of TZJ226 and TZJ637 were 15,068 nucleotides (nt) in length, excluding the poly (A) tail (Supplementary Table S6). The nucleotide homology between the two strains was approximately 99.7%, and combined with ORF5 nucleotide homology, these results genetically indicated that the different PRRSV-1 isolates from this pig farm evolved from the same strain. To determine the phylogenetic relationship of the isolates from this farm with other PRRSV-1 isolates, a whole-genome-based phylogenetic tree was constructed based on all 98 available PRRSV-1 genomes (Figure 2B). The phylogenetic tree showed that the farm PRRSV-1 strain still belonged to BJEU06-1-like according to genome-wide typing. BLAST searches of TZJ226 and TZJ637 in the NCBI database showed the highest consistency with BJEU06-1 (GU047344.1). Compared with the BJEU06-1 strain, their nucleotide identities were 88.4 and 88.5%, representing reductions in homology compared to the previous results for this branch (89.5–93.8%). These results indicated that the BJEU06-1-like breach in China experienced a great deal of variation at the whole-genome level. Combined with the above ORF5 analysis, the homology of the BJEU06-1-like branch was observed to be further reduced, and the results showed that PRRSV-1 is undergoing rapid evolution in China.

**Figure 3:**
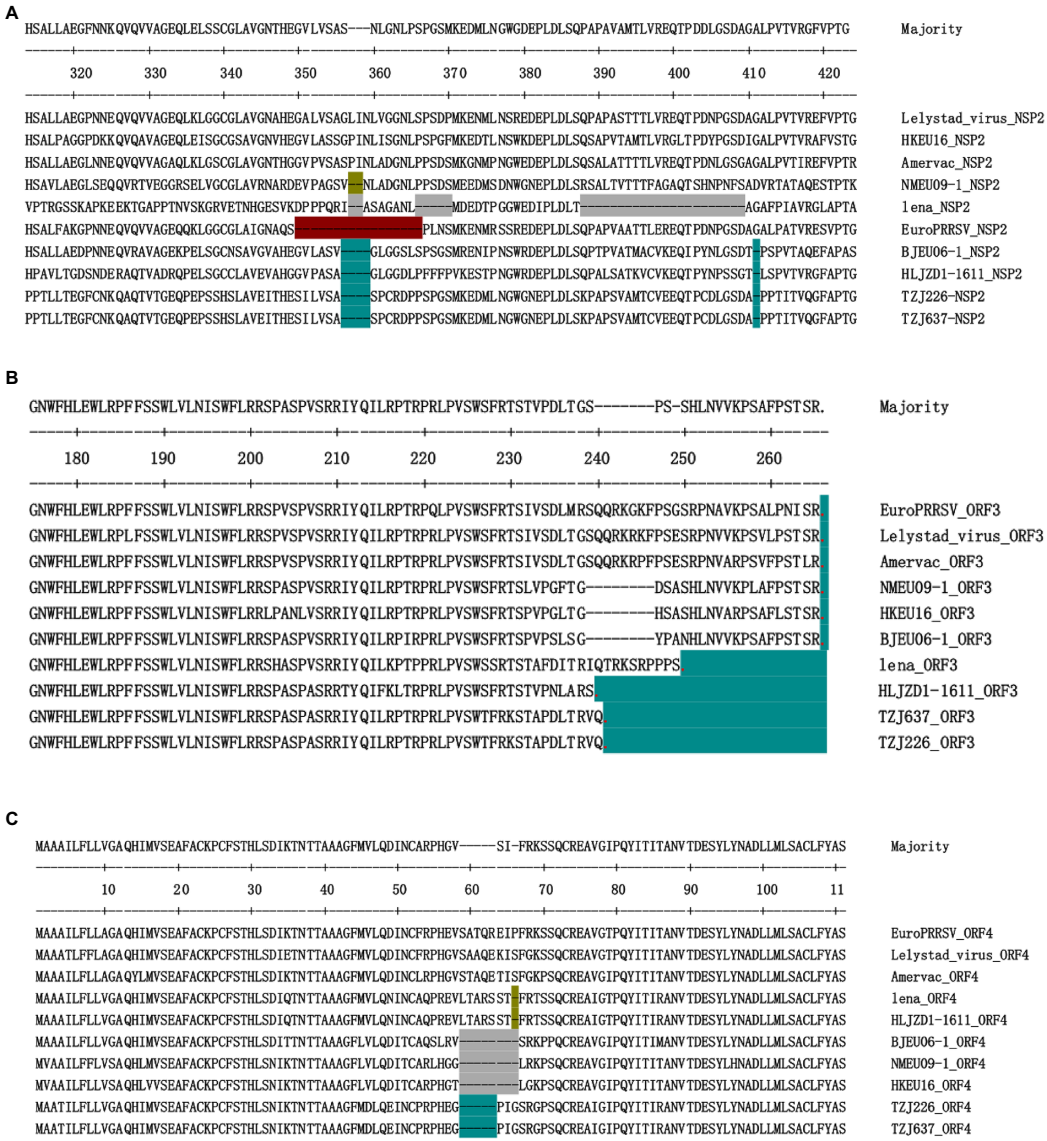
Identification of PRRSV strains with amino acid deletions in NSP2, ORF3 and ORF4. **(A)** Sequence alignment of NSP2 proteins, different aa deletion patterns are indicated by different colors. Similar to the BJEU06-1 strain, TZJ226 and TZJ637 PRRSV show deletions of 4 amino acids and 1 amino acid, respectively, corresponding to positions 356–359 and 411 of the Lelystad NSP2 protein. **(B)** Sequence alignment of ORF3 proteins. Stop codons are represented by red dots, and the number of amino acids lacking due to premature termination relative to Lelystad is indicated by an azure background. **(C)** Sequence alignment of ORF4 proteins, different aa deletion patterns are indicated by different colors. TZJ226 and TZJ637 shared the same 5-aa deletion, labeled with an azure background, corresponding to positions 59–63 of the Lelystad NSP2 protein. The positions marked in the figure represent the positions of the amino acid sequences and refer to the positions in Lelystad.

Each fragment of the TZJ226 and TZJ637 genomes was compared with eight representative strains, and characteristic changes were identified in NSP2, GP3 and GP4 (Figure 3). TZJ226 and TZJ637 are similar to the BJEU06-1-like branch, with a 4 amino acids deletion between aa 356 and 359 and a 1 amino acids deletion at position 411 of NSP2 (Figure 3A). The highly variable PRRSV protein NSP2 is the least conserved viral protein (Allende et al., 1999). Previous molecular epidemiological studies of PRRSV have shown that NSP2 sequences with specific amino acid deletions can easily become characteristic sequences of dominant strains or local epidemic strains, as found in HP-PRRSV, NADC30-like PRRSV and NADC34-like PRRSV (Tong et al., 2007; Zhao et al., 2015; Xu et al., 2020). Hence, our findings are useful for understanding the epidemiological changes in PRRSV-1.

Among the structural proteins of PRRSV-1, ORF3 and ORF4 contain hypervariable regions similar to those of NSP2 (Supplementary Table S6); these hypervariable regions include aa237 to 252 of ORF3 and aa57 to 72 of ORF4. The structural protein encoded by ORF3 in these two strains showed premature termination (Figure 3B): ORF3 was found to be terminated 25 aa early in the TZJ226 and TZJ637 strains in this study. This study is the first to report the 25-aa premature termination of ORF3 of PRRSV-1. The consecutive deletion of 5 aa from positions 59 to 63 of ORF4 was found in TZJ226 and TZJ637 (Figure 3C). In regard to amino acid sequence analysis, the hypervariable region of GP4 extended from 49 to 72 aa; in particular, the 57–68-aa region was revealed to represent the core site of the neutralizing antibody in earlier studies (Vanhee et al., 2010, 2011). Importantly, PRRSV GP4 is a major determinant of viral cellular tropism (Tian et al., 2012). Previous studies have shown that GP4 interacts with CD163, an indispensable receptor for PRRSV infection (Das et al., 2010; Tian et al., 2012). Here, we provide more data to facilitate the study of its biological function. A previous report indicated that ORF3/4 deletion mutants probably evolved from deletion mutant progenitors originating in the Danish epidemic (Chen et al., 2017). It has been reported that PRRSV-1 reported in China may be the product of ancestral isolates introduced from continental Europe that spread to different regions of China and independently experienced mutational accumulation (Chen et al., 2017).

### Recombination analysis

To determine whether recombination events played a role in the generation of the TZJ226 and TZJ637 isolates, RDP4 analysis was performed based on the multiple alignment of 98 PRRSV-1 genomes, and no obvious recombination was found.

## Conclusion

In summary, PRRSV-1 was first reported as one of the main endemic strains on a pig farm where multiple PRRSV subtypes coexist. The PRRSV-1 strains of this farm belong to the BJEU06-1-like branch of subtype 1, and this clade presents high strain diversity. Whole-genome analysis revealed that the same 4 + 1 aa deletion signature found in BJEU06-1-like is present in NSP2 and identified two novel signatures, a 25-aa premature termination in ORF3 and the consecutive deletion of 5 aa in ORF4. Therefore, we should prioritize the continuous monitoring of PRRSV-1 and the strengthening of PRRSV prevention and control measures.

## Data availability statement

The datasets presented in this study can be found in online repositories. The names of the repository/repositories and accession number(s) can be found at: https://www.ncbi.nlm.nih.gov/genbank/, OP566682; https://www.ncbi.nlm.nih.gov/genbank/, OP566683.

## Funding

This study was supported by grants from the National Natural Science Foundation of China (grant nos. 32002315 and 32172890), the China Postdoctoral Fund (grant no. 2020M680788), the Key Programme Foundation of Higher Education of Educational Commission of Henan Province (22A230016) and the National Center of Technology Innovation for Pigs.

## Author contributions

GZ, HZ, LX, and Z-JT: conceptualization. CLi, HX, QS, and JZ: data curation. WL, JL, ZG, QW, CLe, JP, Y-dT, GZ, and JZ: sample collection. CLi and HX: writing—original draft preparation. CLi and JZ: writing—review and editing. Z-JT, BG, TA, and XC: supervision. GZ, HZ, and Z-JT: project administration. All authors have read and agreed to the published version of the manuscript.

## Conflict of interest

The authors declare that the research was conducted in the absence of any commercial or financial relationships that could be construed as a potential conflict of interest.

## Publisher’s note

All claims expressed in this article are solely those of the authors and do not necessarily represent those of their affiliated organizations, or those of the publisher, the editors and the reviewers. Any product that may be evaluated in this article, or claim that may be made by its manufacturer, is not guaranteed or endorsed by the publisher.

## Footnotes

^1^ http://www.datamonkey.org/
