# Supplementary material for: Epidemiological investigation and genetic evolutionary analysis of PRRSV-1 on a pig farm in China
Source: Front Microbiol. 2022 Dec 1;13:1067173. doi: 10.3389/fmicb.2022.1067173 (PMC9751794; doi:10.3389/fmicb.2022.1067173)
Supplement: Supplementary file 1 [file Table_1.DOCX]

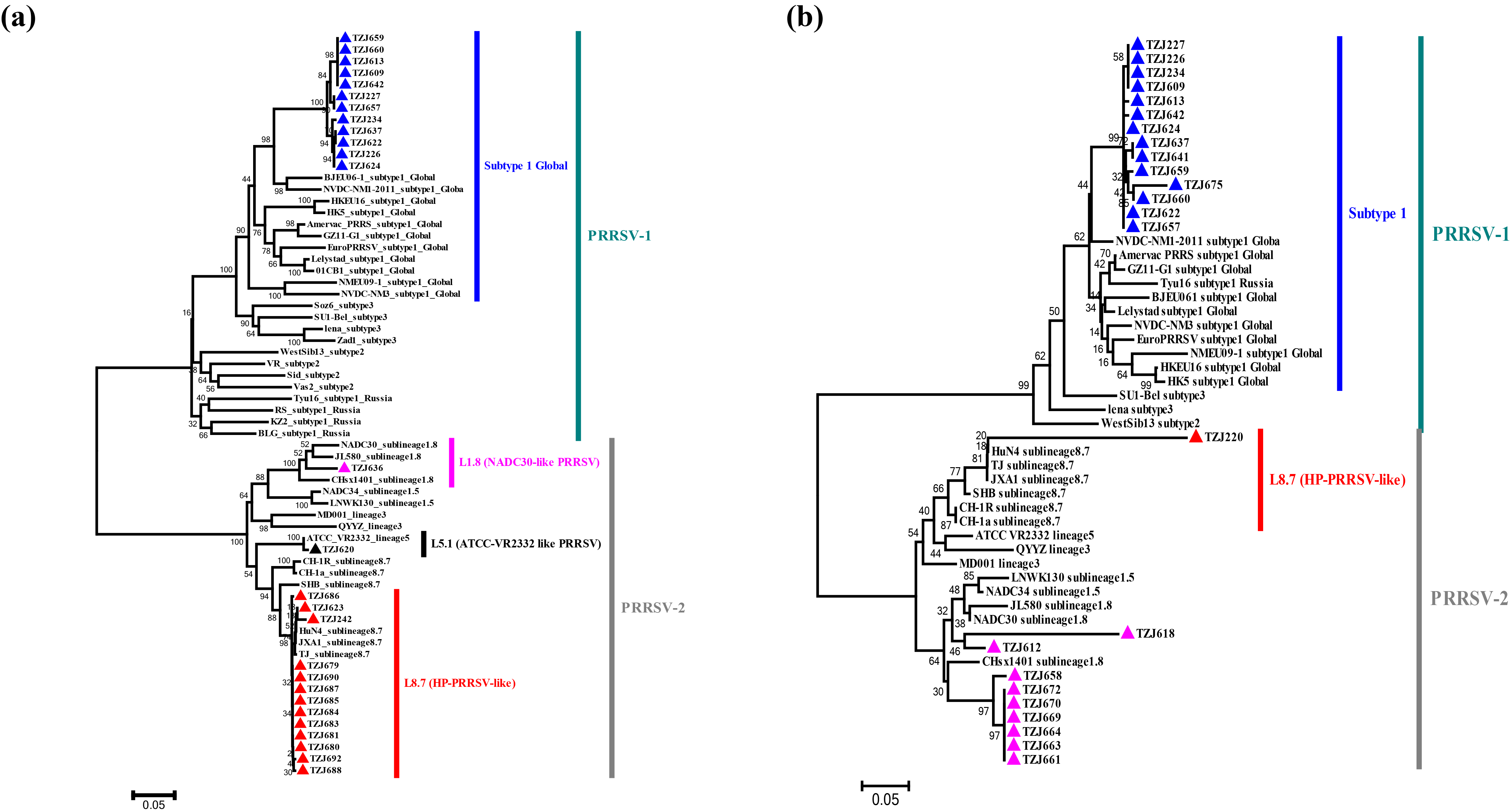


FIG S1. Phylogenetic analysis of PRRSV isolates from the investigated pig producer. (a) Phylogenetic tree constructed based on the ORF5 genes of PRRSV isolates and reference PRRSV strains from each lineage. (b) Phylogenetic tree constructed based on the partial ORF7 gene sequences of PRRSV isolates and reference PRRSV strains from each lineage. PRRSV strains from this farm are indicated by triangles, with different colors representing different lineages.
