# Supplementary material for: Epidemiological investigation and genetic evolutionary analysis of PRRSV-1 on a pig farm in China
Source: Front Microbiol. 2022 Dec 1;13:1067173. doi: 10.3389/fmicb.2022.1067173 (PMC9751794; doi:10.3389/fmicb.2022.1067173)
Supplement: Supplementary file 3 [file Table_3.DOCX]

TABLE S2. Primers used for detection of PRRSV and the amplification of its full-length genome

| Fragment | Primer sequence (5’-3’) | Position in genome | Product size (bp) |
| --- | --- | --- | --- |
| PRRSV-NSP2^a^ | \| ACACCTCCTTTGATTGGGATG \| \| --- \| \| AGTATTTTGGGCGCGTGATCT \| | 2186-3160 | 974 |
| PRRSV-1-ORF5^a^ | AAGTTATCTTTGGGAACGTCTC  GACACCTTAAGGGCATATATCA | 13327-14164 | 838 |
| PRRSV-2-ORF5^a^ | AGCCTGTCTTTTTGCCATTCTG  TCATCACTGGCGTGTAGGTAAT | 13310-14000 | 690 |
| PRRSV-N^a^ | TGCTAGGCCGCAAGTACAT  GGCCGTTGTTATTTGGCATA | 14,581-15,053 | 473 |
| Ly-A^b^ | CCTAGCGTCTGCTTACAGACTACC  AACGCCCCTGGGACACCACATA | 1847-4035 | 2189 |
| Ly-B^b^ | TCCCCTCTGACTCCGTACAAC  AACGCCCCTGGGACACCACATA | 1563-4035 | 2473 |
| Ly-C^b^ | CAGCGCCAACTTTGGGAACCTG  CACAAAAGTTGAACGGTCGAGA | 3861-5923 | 2063 |
| Ly-D^b^ | TTGGTTCTGGTCTTGTGACAAC  TGGATTATTTGCTTGGATAACTC | 5698-7764 | 2067 |
| Ly-E^b^ | GTGGAGGTAAAGAAATCAACTGA  AGCCACCTTCACCATGTTTAT | 7523-9727 | 2205 |
| Ly-F^b^ | GGAGGTACCAGTCCCGTCGAGG  GGCTGTTGCCGGTCCTATACAC | 9591-11410 | 1820 |
| Ly-G^b^ | AGTTGGAAGGGCTCACGTGGTC  AGGCGAACGCCTCAGAAACC | 11304-12997 | 1694 |
| Ly-H^b^ | TATTATCACCACCAAATAGACGG  CTTCTCAGGCTTTTTCCTTTT | 12887-14740 | 2196 |

^a^ Primers used for detection of PRRSV; ^b^ Primers used for amplification of the whole genome of PRRSV-1. The primer sequences, position in the genome and product size were based on the Lelystad (M96262.2) strain.
