# Supplementary material for: Epidemiological investigation and genetic evolutionary analysis of PRRSV-1 on a pig farm in China
Source: Front Microbiol. 2022 Dec 1;13:1067173. doi: 10.3389/fmicb.2022.1067173 (PMC9751794; doi:10.3389/fmicb.2022.1067173)
Supplement: Supplementary file 4 [file Table_4.DOCX]

TABLE S3. PRRSV reference genomes used in this study

| No. | Designated name | Country  of origin | Year isolated | Accession no |
| --- | --- | --- | --- | --- |
| 1 | AUT13-883 | Austria | 2013 | KT326148.1 |
| 2 | Austria | Austria | 2014 | KT334375.1 |
| 3 | lena | Belarus | 2007 | JF802085.1 |
| 4 | SU1-Be | Belarus | 2010 | KP889243.1 |
| 5 | Zad-1 | Belarus | none | DQ324694.2 |
| 6 | Soz-6 | Belarus | none | DQ324686.2 |
| 7 | Vas-2 | Belarus | none | DQ324689.1 |
| 8 | 07V063 | Belgium | 2007 | GU737264.2 |
| 9 | 13V117 | Belgium | 2013 | KT159249.1 |
| 10 | 13V091 | Belgium | 2013 | KT159248.1 |
| 11 | DK-1992-PRRS-111_92 | Denmark | 1992 | KC862566.1 |
| 12 | DK-2003-6-5 | Denmark | 2003 | KC862571.1 |
| 13 | DK-2008-10-5-2 | Denmark | 2008 | KC862573.1 |
| 14 | DK-2010-10-10-3 | Denmark | 2010 | KC862568.1 |
| 15 | DK-2011-05-23-9 | Denmark | 2011 | KC862569.1 |
| 16 | DK-2011-05-11-14 | Denmark | 2011 | KC862567.1 |
| 17 | DK-2012-01-05-2 | Denmark | 2012 | KC862574.1 |
| 18 | PRRS-FR-2005-29-24-1 | France | 2005 | KY366411.1 |
| 19 | PRRS-FR-2014-56-11-1 | France | 2014 | KY767026.1 |
| 20 | GER09-613 | Germany | 2009 | KT344816.1 |
| 21 | 14432/2011 | Hungary | 2011 | KR296711.1 |
| 22 | 9625/2012 | Hungary | 2012 | KJ415276.1 |
| 23 | Sid | Lithuania | none | DQ324682.1 |
| 24 | Lelystad | Netherlands | 1993 | M96262.2 |
| 25 | DV | Netherlands | 1996 | KF991509.2 |
| 26 | LV4.2.1 | Netherlands | 2004 | AY588319.1 |
| 27 | Cresa3267 | Portugal | 2006 | JF276435.1 |
| 28 | WestSib13 | Russia | 2013 | KX668221.1 |
| 29 | Tyu16 | Russia | 2016 | MT008024.1 |
| 30 | VR | Russia | none | EU071233.1 |
| 31 | KZ-2 | Russia | none | EU071239.1 |
| 32 | BLG | Russia | none | EU071232.1 |
| 33 | RS | Russia | none | EU071230.1 |
| 34 | Olot/91 | Spain | 1991 | KF203132.1 |
| 35 | Cresa3262 | Spain | 1992 | JF276431.1 |
| 36 | MLV-DV | Spain | 1999 | KJ127878.1 |
| 37 | Cresa3249 | Spain | 2005 | JF276433.1 |
| 38 | Cresa3256 | Spain | 2005 | JF276432.1 |
| 39 | CReSA228 | Spain | 2013 | KX249755.1 |
| 40 | CReSA3 | Spain | 2013 | KX249748.1 |
| 41 | CReSA184 | Spain | 2013 | KX249754.1 |
| 42 | CReSA261 | Spain | 2013 | KX249756.1 |
| 43 | CReSA46 | Spain | 2014 | KX249751.1 |
| 44 | CReSA70 | Spain | 2014 | KX249752.1 |
| 45 | CReSA38 | Spain | 2014 | KX249750.1 |
| 46 | CReSA17 | Spain | 2014 | KX249749.1 |
| 47 | CReSA100 | Spain | 2014 | KX249753.1 |
| 48 | Amervac | Spain | Vaccine | GU067771.1 |
| 49 | IVI-1173 | Switzerland | 2012 | KX622783.1 |
| 50 | 195-05 | United Kingdom | 2005 | KU560579.1 |
| 51 | SD01-08 | USA | 2001 | DQ489311.1 |
| 52 | EuroPRRSV | USA | 2003 | AY366525.1 |
| 53 | SD03-15_P83 | USA | 2003 | KU131560.1 |
| 54 | 94881 | USA | 2006 | KT988004.1 |
| 55 | USA/ISU02609/2010 (EU01) | USA | 2010 | MK359258.1 |
| 56 | USA/ISU01969/2011 (EU02) | USA | 2011 | MK359259.1 |
| 57 | USA/ISU00731/2012 (EU03) | USA | 2012 | MK359260.1 |
| 58 | USA/ISU43354/2012 (EU04) | USA | 2012 | MK359261.1 |
| 59 | USA/ISU11271/2013 (EU05) | USA | 2013 | MK359262.1 |
| 60 | USA/ISU78021/2015 (EU06) | USA | 2015 | MK359263.1 |
| 61 | USA/ISU37369/2017 (EU07) | USA | 2017 | MK359264.1 |
| 62 | USA/ISU41597/2017 (EU08) | USA | 2017 | MK359265.1 |
| 63 | USA/ISU58061/2017 (EU09) | USA | 2017 | MK359266.1 |
| 64 | USA/ISU79359/2017(EU10) | USA | 2017 | MK359267.1 |
| 65 | USA/ISU85615/2017 (EU11) | USA | 2017 | MK359268.1 |
| 66 | USA/ISU38853/2017 (EU12) | USA | 2017 | MK359269.1 |
| 67 | USA/ISU39201/2017 (EU13) | USA | 2017 | MK359270.1 |
| 68 | USA/ISU44362/2017 (EU14) | USA | 2017 | MK359271.1 |
| 69 | USA/ISU41593/2017 (EU15) | USA | 2017 | MK359272.1 |
| 70 | USA/ISU50779/2017 (EU16) | USA | 2017 | MK359273.1 |
| 71 | USA/ISU55989/2017 (EU17) | USA | 2017 | MK359274.1 |
| 72 | USA/ISU57212/2017 (EU18) | USA | 2017 | MK359275.1 |
| 73 | USA/ISU79610/2017 (EU19) | USA | 2017 | MK359276.1 |
| 74 | USA/ISU81436/2017 (EU20) | USA | 2017 | MK359277.1 |
| 75 | USA/ISU82347/2017 (EU21) | USA | 2017 | MK359278.1 |
| 76 | USA/ISU86456/2017 (EU22) | USA | 2017 | MK359279.1 |
| 77 | USA/ISU00669/2018 (EU23) | USA | 2018 | MK359280.1 |
| 78 | USA/ISU21886/2018 (EU24) | USA | 2018 | MK359281.1 |
| 79 | USA/ISU21775/2018 (EU25) | USA | 2018 | MK359282.1 |
| 80 | USA/ISU20054/2018 (EU26) | USA | 2018 | MK359283.1 |
| 81 | USA/ISU60533/2018 (EU27) | USA | 2018 | MK359284.1 |
| 82 | 01CB1 | Thailand | 2001 | DQ864705.1 |
| 83 | E38 | South Korea | 2007 | KT033457.1 |
| 84 | KNU-07 | South Korea | 2007 | FJ349261.1 |
| 85 | EuroViet-01 | Vietnam | 2016 | MG251834.1 |
| 86 | EuroViet-02 | Vietnam | 2016 | MG251833.1 |
| 87 | EuroViet-03 | Vietnam | 2016 | MG251835.1 |
| 88 | B13 | China | 1999 | AY633973.1 |
| 89 | HK3 | China | 2003 | KF287129.1 |
| 90 | HK5 | China | 2004 | KF287130.1 |
| 91 | HK8 | China | 2004 | KF287128.1 |
| 92 | HK10 | China | 2004 | KF287131.1 |
| 93 | BJEU06-1 | China | 2006 | GU047344.1 |
| 94 | FJ0603 | China | 2006 | HM114313.1 |
| 95 | FJ0602 | China | 2006 | HM755885.1 |
| 96 | HKEU16 | China | 2007 | EU076704.1 |
| 97 | NMEU09-1 | China | 2009 | GU047345.1 |
| 98 | SHE | China | 2009 | GQ461593.1 |
| 99 | NVDC-NM1-2011 | China | 2011 | JX187609.1 |
| 100 | GZ11-G1 | China | 2011 | KF001144.1 |
| 101 | NVDC-FJ | China | 2011 | KC492506.1 |
| 102 | NVDC-NM2 | China | 2011 | KC492504.1 |
| 103 | NVDC-NM3 | China | 2011 | KC492505.1 |
| 104 | LNEU12 | China | 2012 | KM196101.1 |
| 105 | FJEU13 | China | 2013 | KP860912.1 |
| 106 | FJQEU14 | China | 2014 | KP860913.1 |
| 107 | HLJB1 | China | 2014 | KT224385.1 |
| 108 | 15HEN1 | China | 2015 | KX967492.1 |
| 109 | GDFS16 | China | 2016 | MF153486.1 |
| 110 | GDGZ16 | China | 2016 | MF153487.1 |
| 111 | GDHY16 | China | 2016 | MF153488.1 |
| 112 | GDHZ16 | China | 2016 | MF153489.1 |
| 113 | GDJM16 | China | 2016 | MF153490.1 |
| 114 | GDJY16 | China | 2016 | MF153491.1 |
| 115 | GDMM16 | China | 2016 | MF153492.1 |
| 116 | GDMZ16 | China | 2016 | MF153493.1 |
| 117 | GDQY16 | China | 2016 | MF153494.1 |
| 118 | GDSG16 | China | 2016 | MF153495.1 |
| 119 | GDSW16 | China | 2016 | MF153496.1 |
| 120 | GDYF16 | China | 2016 | MF153497.1 |
| 121 | GDYJ16 | China | 2016 | MF153498.1 |
| 122 | GDZJ16 | China | 2016 | MF153499.1 |
| 123 | GDZQ16 | China | 2016 | MF153500.1 |
| 124 | HENZMD-10 | China | 2017 | KY363382.1 |
| 125 | KZ2018 | China | 2018 | MN550991.1 |
| 126 | NPUST-2789-3W-2 | China | 2018 | MN242825.1 |
| 127 | NPUST-2789-3W-5 | China | 2018 | MN265857.1 |
| 128 | NPUST-2860-S-6 | China | 2018 | MN265858.1 |
| 129  130  131 | HN1804–1  TZJ226  TZJ637 | China  China  China | 2018  2020  2020 | MK689121.1  OP566682.1  OP566683.1 |
