# Supplementary material for: Epidemiological investigation and genetic evolutionary analysis of PRRSV-1 on a pig farm in China
Source: Front Microbiol. 2022 Dec 1;13:1067173. doi: 10.3389/fmicb.2022.1067173 (PMC9751794; doi:10.3389/fmicb.2022.1067173)
Supplement: Supplementary file 5 [file Table_5.DOCX]

TABLE S4. ORF5 nucleotide sequence identity on the BJEU06-1-like branch

| Strain | Identity (%) | | | | | | |
| --- | --- | --- | --- | --- | --- | --- | --- |
|  | JX187609.1_NVDC-NM1-2011 | KM196101.1_LNEU12 | KY363382.1_HENZMD-10 | GU047344.1_BJEU06-1 | KP860912.1_FJEU13 | MN550991.1_KZ2018 | KX967492.1_15HEN1_EU |
| This farm | 88.3-88.8% | 86.8-87.3% | 86.6-87.1% | 87.9-88.6% | 85.8-86.3% | 85.0-85.6% | 87.0-87.8% |
| JX187609.1_NVDC-NM1-2011 | - | 94.22 | 90.59 | 92.74 | 90.59 | 90.26 | 90.26 |
| KM196101.1_LNEU12 | 94.22 | - | 90.59 | 91.58 | 90.92 | 89.6 | 90.59 |
| KY363382.1_HENZMD-10 | 90.59 | 90.59 | - | 89.6 | 86.3 | 85.15 | 86.96 |
| GU047344.1_BJEU06-1 | 92.74 | 91.58 | 89.6 | - | 89.93 | 89.77 | 90.92 |
| KP860912.1_FJEU13 | 90.59 | 90.92 | 86.3 | 89.93 | - | 89.44 | 89.93 |
| MN550991.1_KZ2018 | 90.26 | 89.6 | 85.15 | 89.77 | 89.44 | - | 87.46 |
| KX967492.1_15HEN1_EU | 90.26 | 90.59 | 86.96 | 90.92 | 89.93 | 87.46 | - |
