# Supplementary material for: Epidemiological investigation and genetic evolutionary analysis of PRRSV-1 on a pig farm in China
Source: Front Microbiol. 2022 Dec 1;13:1067173. doi: 10.3389/fmicb.2022.1067173 (PMC9751794; doi:10.3389/fmicb.2022.1067173)
Supplement: Supplementary file 7 [file Table_7.DOCX]

TABLE S6 Detailed complete genome comparison of TZJ226 and TZJ637 with representative PRRSV 1 strains.

| Region | Length^a^ | | Similarity to TZJ226/TZJ637 (%) | | | | |
| --- | --- | --- | --- | --- | --- | --- | --- |
| Nucleotides (nt) | TZJ226 | TZJ637 | LV | Amervac | HKEU16 | BJEU06-1 | NMEU09-1 |
| 5′UTR | 221 | 221 | 95.0/95.0 | 95.5/95.5 | 94.6/94.6 | 94.6/94.6 | 95.9/95.9 |
| ORF1a | 7173 | 7173 | 86.4/86.3 | 84.6/84.5 | 83.0/82.9 | 86.9/86.9 | 79.9/79.8 |
| ORF1b | 4386 | 4386 | 100/99.6 | 88.2/88.3 | 86.8/86.8 | 88.2/88.3 | 85.0/85.1 |
| ORFs2-7 | 3168 | 3168 | 88.9/88.8 | 87.6/87.6 | 84.8/85.6 | 86.4/88.5 | 84.4/84.2 |
| 3′UTR | 114 | 114 | 92.1/92.2 | 91.2/91.2 | 90.4/90.4 | 93.9/93.9 | 92.1/92.1 |
| Complete | 15068 | 15068 | 88.0/88.0 | 86.5/86.5 | 85.1/85.1 | 88.5/88.6 | 82.9/82.9 |
| Proteins (AA) | | | | | | | |
| Nsp1α | 180 | 180 | 92.2/91.7 | 90.6/90.6 | 85.2/84.8 | 90.6/90.0 | 90.6/90.0 |
| Nsp1β | 205 | 205 | 80.5/80.5 | 78.5/78.5 | 80.0/80.0 | 88.3/88.3 | 76.6/76.6 |
| Nsp2 | 1073 | 1073 | 82.0/81.9 | 80.2/80.1 | 78.3/78.4 | 82.6/82.5 | 73.4/73.5 |
| Nsp3 | 230 | 230 | 93.5/93.5 | 93.5/93.5 | 92.2/92.2 | 93.5/93.5 | 93.9/93.9 |
| Nsp4 | 203 | 203 | 92.1/92.1 | 100/100 | 91.1/91.1 | 93.1/93.1 | 84.7/84.7 |
| Nsp5 | 170 | 170 | 92.9/91.8 | 92.9/91.8 | 88.2/87.1 | 84.1/92.9 | 87.6/86.5 |
| Nsp6 | 16 | 16 | 100/100 | 100/100 | 93.8/93.8 | 100/100 | 100/100 |
| Nsp7α | 149 | 149 | 91.3/91.1 | 89.5/89.3 | 87.7/87.5 | 91.1/90.8 | 87.7/87.5 |
| Nsp7β | 120 | 120 | 93.3/93.9 | 93.3/93.3 | 94.2/94.2 | 93.3/93.3 | 90.0/90.0 |
| Nsp8 | 45 | 45 | 91.1/91.1 | 88.9/88.9 | 84.4/84.4 | 93.3/93.3 | 86.7/86.7 |
| Nsp9 | 645 | 645 | 96.9/97.2 | 96.9/97.2 | 95.7/96.0 | 96.6/96.9 | 95.0/95.3 |
| Nsp10 | 442 | 442 | 95.0/94.8 | 94.8/94.6 | 91.9/91.6 | 95.9/95.7 | 94.1/93.9 |
| Nsp11 | 224 | 224 | 96.9/96.9 | 96.9/96.9 | 95.5/95.5 | 97.8/97.8 | 95.5/95.5 |
| Nsp12 | 152 | 152 | 92.9/92.3 | 94.9/94.2 | 91.7/91.0 | 92.1/91.4 | 92.3/91.7 |
| GP2a | 250 | 250 | 91.2/90.8 | 87.2/87.6 | 87.2/87.6 | 92.8/93.2 | 86.0/86.4 |
| E | 71 | 71 | 94.4/94.4 | 94.4/94.4 | 93.3/93.3 | 97.2/97.2 | 97.2/97.2 |
| GP3 | 261 | 261 | 89.0/89.0 | 88.3/88.3 | 85.9/85.9 | 88.8/88.8 | 85.5/85.5 |
| GP4 | 179 | 179 | 88.3/88.3 | 88.8/88.8 | 89.2/89.2 | 90.3/90.3 | 89.2/89.2 |
| GP5 | 202 | 202 | 86.6/87.1 | 88.1/88.6 | 87.1/87.6 | 86.6/86.6 | 84.7/84.7 |
| GP5a | 44 | 44 | 95.5/95.5 | 93.2/93.2 | 93.2/93.2 | 88.6/88.6 | 95.5/95.5 |
| M | 174 | 174 | 96.6/97.1 | 96.0/96.6 | 93.1/93.7 | 92.5/93.1 | 92.5/93.1 |
| N | 129 | 129 | 91.5/90.7 | 90.7/89.9 | 86.0/85.3 | 90.7/89.9 | 86.8/86.0 |

^a^ The length of each fragment/protein in the TZJ226 and TZJ637 genomes.
